# Supplementary material for: Synergistic effect of CNTF and GDNF on directed neurite growth in chick embryo dorsal root ganglia
Source: PLoS One. 2020 Oct 5;15(10):e0240235. doi: 10.1371/journal.pone.0240235 (PMC7535060; doi:10.1371/journal.pone.0240235)
Supplement: S1 File — (PDF) [file pone.0240235.s001.pdf]

Identifying Candidate Neurotrophic Factors for the Guidance Assay

We initially selected six factors based on the analysis of the available literature [1 – 11], including CNTF, GDNF, NGF, IGF, FGF, and BDNF. Before proceeding with the guidance assay, we first tested all six candidate factors individually and in pair-wise combinations in a homogenous medium to establish which of them would be most efficient in stimulating the overall outgrowth of neuronal processes in chick embryonic DRGs, regardless of the direction of the growth.

In the first series of experiments, we tested the neurotrophic factors individually. In this experimental setup, individual DRGs were co-cultured with mouse myoblasts (C2C12) for 3 days in media, all of which contained DMEM/HG with 2% horse serum and one of the neurotrophic factors: 250 ng/mL FGF, 10 ng/mL GDNF, 10 ng/mL CNTF, 50 ng/mL BDNF, 50 mg/mL NGF, and 100 ng/mL IGF. After 3 days in culture, the DRGs were fixed and stained with an anti-neurofilament antibody, and the average length of growing axons was quantified for each treatment group. This experiment showed that three factors – CNTF, GDNF, and BDNF – showed superior performance in promoting the neurite outgrowth **(Fig. S1-1)**. These factors were selected for the second experiment, where they were tested in pair-wise combinations to establish whether or not they will exhibit synergistic effects.

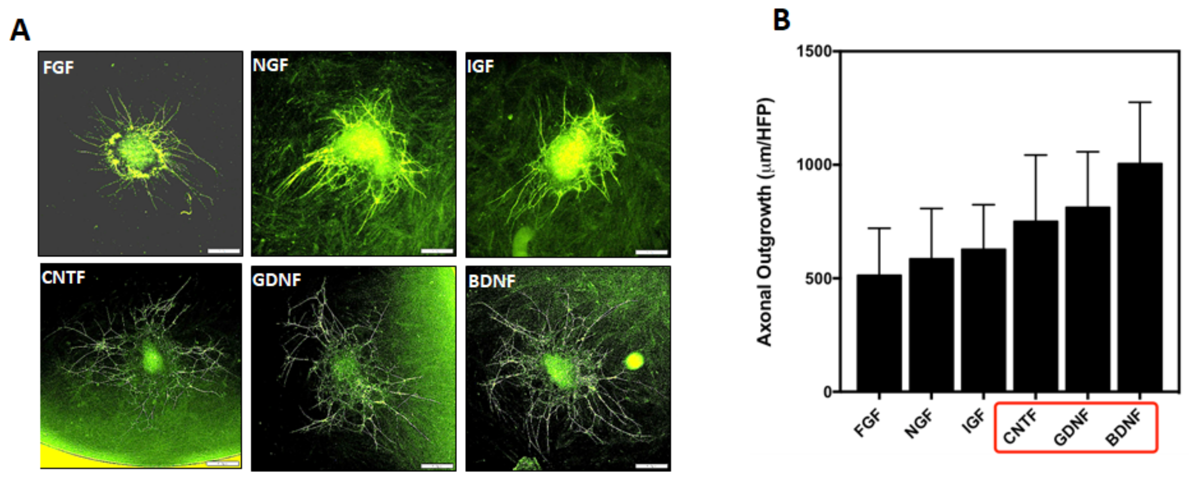

Figure S1-1. Effect of individual neurotrophic factors on neurite outgrowth in DRGs co-cultured with C2C12 myoblasts. **A.** Representative immunofluorescent micrographs of DRGs and outgrowing neurites stained with an anti-neurofilament antibody. Scale bar – 200 μm **B.** Average length of neurites (three DRGs were quantified from each treatment group) Data are plotted as the mean ± standard deviation.

To test the pair-wise combinations of neurotrophic factors, the DRGs were co-cultured with C2C12 myoblasts as above in either the base medium (DMEM/HG with 2% horse serum) in the absence of neurotrophic factors (control), or in the base medium containing GDNF and CNTF (10 ng/mL each), GDNF (10 ng/mL) and BDNF (50 ng/mL), or CNTF (10 ng/mL) and BDNF (50 ng/mL). After 3 days in culture in the respective media, the samples were immunostained and quantified as above. The quantitative analysis of the average neurite length showed that both the GDNF+CNTF and BDNF+GDNF combinations significantly increased the neurite outgrowth as compared to the control group (the base medium), with the GDNF+CNTF treatment being the most efficient. On the other hand, the BDNF+CNTF combination had no significant effect when compared to the control group **(Fig. S1-2).**

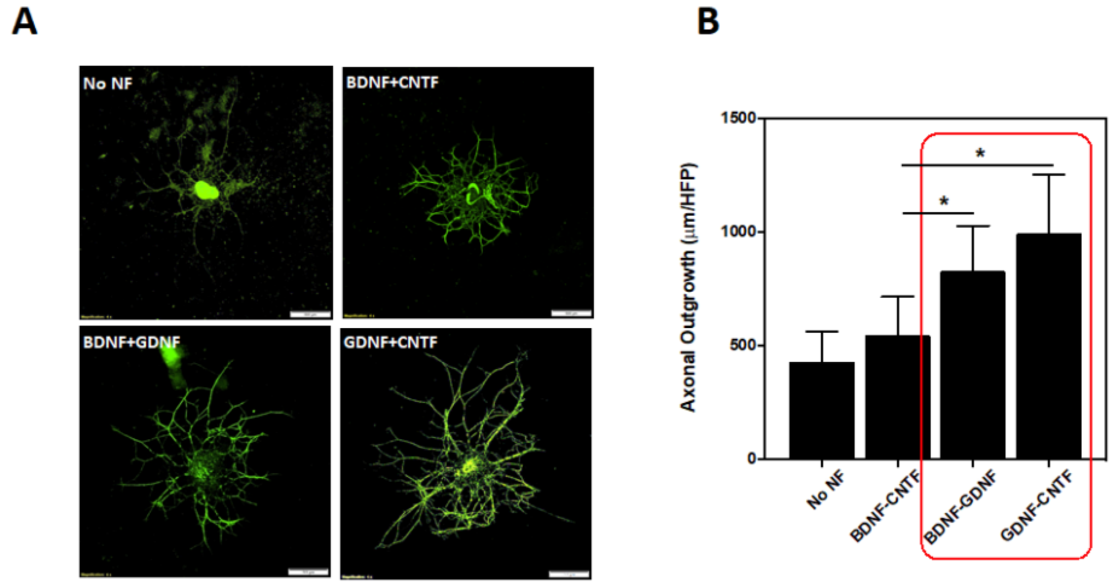

Figure S1-2. Effect of pair-wise combinations of the neurotrophic factors CNTF, GDNF, and BDNF on neurite outgrowth in DRGs co-cultured with myoblasts. **A.** Representative immunofluorescent micrographs of DRGs and outgrowing neurites stained with an anti-neurofilament antibody. Scale bar – 200 μm. **B.** Average length of axons (three DRGs were quantified from each treatment group). Data are plotted as the mean ± standard deviation

Besides measuring the length of the neurites, we also used an alternative method to quantify the neurite outgrowth from the DRGs as the relative area of occupied by neurites in micrographs **(Fig. S1-3A-C)**. This approach takes into account not only the length, but also the width of the neuronal processes. All micrographs had the same dimensions and were taken at the same resolution. The region of the image corresponding to the ganglion body was masked in the ImageJ software (**Fig. S1-3A**). The images were then converted into a binary format (**Fig. S1-3B**), and the number of pixels occupied by neurites growing from the DRG body was calculated using the threshold tool in ImageJ (**Fig. S1-3C**). The quantification data showed a significantly increased relative area of neurites in the CNTF-GDNF group as compared to the control group. None of the other treatment groups showed any significant differences compared with the control (n = 3, ANOVA, Tukey post-hoc test, *$p < 0.05$) (**Fig. S1-3D**).

<u>Conclusion:</u>

The pilot experiments outlined above allowed us to select CNTF and GDNF as the candidate neurotrophic factors for the guidance assay, since their mix had the most stimulatory effect on the neurite outgrowth.

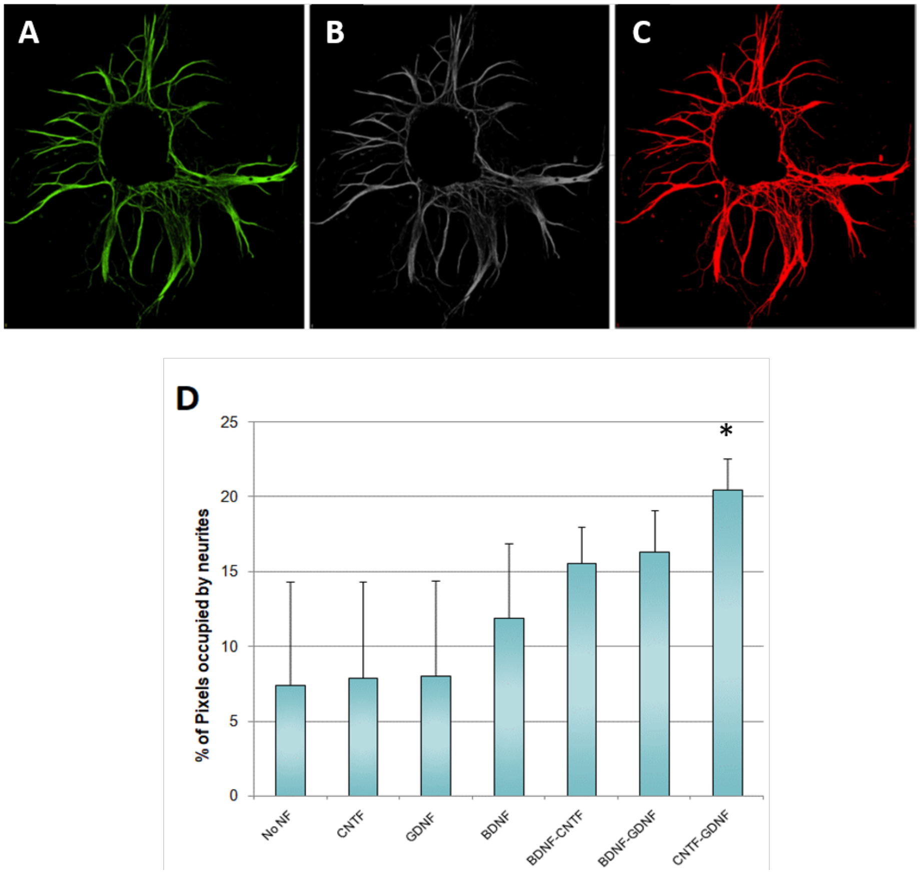

Figure S1-3. Relative area occupied by neurites in micrographs of the DRG explant assay. **A - C.** Image processing to quantify the neurite outgrowth as the relative area occupied by neuronal processes. The samples were processed for immunofluorescent staining with anti-NF antibodies as above. **A.** Exclusion of the DRG body by masking the corresponding region of the image. **B.** Image conversion into a binary format. **C.** Pixels corresponding to neurites are highlighted using a threshold tool. **D.** Bar plot of the relative area of neurites (n = 3, ANOVA and Tukey test, *$p < 0.05$ compared with the control samples growth in the absence of the neurotrophic factors (No NF). Data are plotted as the mean ± standard deviation).

References:

1. Sakuma, K. and A. Yamaguchi, *The recent understanding of the neurotrophin's role in skeletal muscle adaptation.* J Biomed Biotechnol, 2011. **2011**: p. 201696.

2. Vandenburgh, H.H., et al., *Insulin and IGF-I induce pronounced hypertrophy of skeletal myofibers in tissue culture.* Am J Physiol, 1991. **260**(3 Pt 1): p. C475-84.

3. Florini, J.R., et al., *Spontaneous Differentiation of Skeletal Myoblasts Is Dependent Upon Autocrine Secretion of Insulin-Like Growth Factor-Ii.* Journal of Biological Chemistry, 1991. **266**(24): p. 15917-15923.

4. Apel, P.J., et al., *Effect of locally delivered IGF-1 on nerve regeneration during aging: an experimental study in rats.* Muscle Nerve, 2010. **41**(3): p. 335-41.

5. Rende, M., et al., *Nerve growth factor (NGF) influences differentiation and proliferation of myogenic cells in vitro via TrKA.* Int J Dev Neurosci, 2000. **18**(8): p. 869-85.

6. Baron, P., et al., *Expression of the low-affinity NGF receptor during human muscle development, regeneration, and in tissue culture.* Muscle Nerve, 1994. **17**(3): p. 276-84.

7. Bathina, S. and U.N. Das, *Brain-derived neurotrophic factor and its clinical implications.* Arch Med Sci, 2015. **11**(6): p. 1164-78.

8. Li, Q., et al., *Nerve conduit filled with GDNF gene-modified Schwann cells enhances regeneration of the peripheral nerve.* Microsurgery, 2006. **26**(2): p. 116-21.

9. Marques, M.J. and H.S. Neto, *Ciliary neurotrophic factor stimulates in vivo myotube formation in mice.* Neurosci Lett, 1997. **234**(1): p. 43-6.

10. Bach, A.D., J.P. Beier, and G.B. Stark, *Expression of Trisk 51, agrin and nicotinic-acetycholine receptor epsilon-subunit during muscle development in a novel three-dimensional muscle-neuronal co-culture system.* Cell Tissue Res, 2003. **314**(2): p. 263-74.

11. Peroulakis, M.E. and N.G. Forger, *Ciliary neurotrophic factor increases muscle fiber number in the developing levator ani muscle of female rats.* Neurosci Lett, 2000. **296**(2-3): p. 73-6.
